# Participants in the Trans-Antarctic Winter Traverse Expedition Showed Increased Bacterial Load and Diversity in Saliva but Maintained Individual Differences within Stool Microbiota and Across Metabolite Fingerprints

**DOI:** 10.3390/ijms24054850

**Published:** 2023-03-02

**Authors:** Simon J. S. Cameron, Arwyn Edwards, Robert J. Lambert, Mike Stroud, Luis A. J. Mur

**Affiliations:** 1 Institute for Global Food Security, School of Biological Sciences, Queen’s University Belfast, Chlorine Gardens, Belfast BT9 5DL, UK; 2 Institute of Biological, Environmental and Rural Sciences, Edward Llywd Building, Penglais Campus, Aberystwyth SY23 3FG, UK; 3 Department of Orthopaedics, Royal Infirmary of Edinburgh, 51 Little France Crescent, Edinburgh EH16 4SA, UK; 4 NIHR BRC Nutrition, University of Southampton Medical School, Southampton SO16 6YD, UK

**Keywords:** salivary microbiota, stool microbiota, metabolomics, metabolite fingerprinting, isolation, longitudinal, microbiota dynamics, microbiota-metabolome integration

## Abstract

Understanding the impact of long-term physiological and environmental stress on the human microbiota and metabolome may be important for the success of space flight. This work is logistically difficult and has a limited number of available participants. Terrestrial analogies present important opportunities to understand changes in the microbiota and metabolome and how this may impact participant health and fitness. Here, we present work from one such analogy: the Transarctic Winter Traverse expedition, which we believe is the first assessment of the microbiota and metabolome from different bodily locations during prolonged environmental and physiological stress. Bacterial load and diversity were significantly higher during the expedition when compared with baseline levels (*p* < 0.001) in saliva but not stool, and only a single operational taxonomic unit assigned to the Ruminococcaceae family shows significantly altered levels in stool (*p* < 0.001). Metabolite fingerprints show the maintenance of individual differences across saliva, stool, and plasma samples when analysed using flow infusion electrospray mass spectrometry and Fourier transform infrared spectroscopy. Significant activity-associated changes in terms of both bacterial diversity and load are seen in saliva but not in stool, and participant differences in metabolite fingerprints persist across all three sample types.

## 1. Introduction

Stress that impacts a person’s normal lifestyle can lead to changes in the human microbiota [1]. Given this, it is likely that extreme stress may have an impact on the human microbiota that affects physiology and endurance in a range of scenarios. It has been suggested, for example, that the stress associated with military combat training increases intestinal permeability and symptoms of gastrointestinal disease [2], which may have important implications for the human microbiota. These factors could also be a major consideration in long-duration space travel by humans and could be important factors in health and fitness screening of potential participants in long-term exploration journeys. The human stress response centres on the endocrine system to produce hormones such as corticosteroids, which exert rapid non-genomic effects on neurons in the hypothalamus to help maintain homeostasis and adapt to stressful stimuli [3]. Stress can also increase the permeability of the gut, potentially allowing the gut microbiota to indirectly influence the human body through the hypothalamic–pituitary–adrenal axis (HPA). The human microbiota has been shown to modulate the host’s stress response through the production or alteration of key neurotransmitter systems, such as the serotonergic (5HT), norepinephrine (NE) and endorphin systems [4]. Indeed, work using animal models has suggested that the microbiota could influence depression [5] or anxiety and stress [6]. Further, the microbiota could play a role in various pathologies, including those involving the central nervous system [7], and could affect CNS auto-immune diseases, such as multiple sclerosis, Devic’s disease, and Guillain–Barré [8]. There have been limited human-focused studies investigating the link between the microbiota and stress and they have been confined to assessing the impact of probiotics [9].

In this current study, our motivation was to examine the impact of prolonged stressful conditions on the human microbiota and metabolome, which may prove relevant to long-duration space travel. To date, the field of medicine that is interested in the effect of long-duration space travel has focused on improving the provision of artificial life-support systems, though the human microbiota and metabolome have received some attention [10]. Nevertheless, the combined effects of isolation and extreme conditions on these human systems are unknown. Considering practical limitations, a substantial amount of work has been completed on the impact of space travel on the environmental microbiota [11,12]. Directly studying the impact of long-duration space travel on the human microbiota is difficult; it is confounded by issues such as very small participant cohorts, sample collection, stable long-term storage, and the intact return of materials to Earth. Effective studies have been completed on animal models and astronaut twin pairings that show that long-term space travel is marked by a mitochondrial stress response [13]. Terrestrial analogues present potential alternative models for space travel, allowing for the study of the human microbiota and metabolome under conditions representative of those that may be encountered. Isolation studies attempting to mimic the effects of prolonged space travel have measured changes in the structure and function of the human gut microbiota. In the Mars-500 study, the crew of volunteers lived and worked in a mock-up spacecraft to simulate a 520-day manned return mission to Mars. Changes in the taxonomic composition of the gut microbiota were seen within the first 14 days but no such changes were seen in its core functional capacity, with a reversion to the initial microbiota composition beginning to be seen within two weeks of the study ending [14]. More recently, additional work has highlighted the potential role of targeted probiotics for the promotion of astronaut health and to alleviate symptoms of illness [15]. Beyond the human microbiota, the broader implications of long-term space travel on health and metabolism are increasingly being explored, alongside potential nutritional counter-measures [16]. A broader appreciation and utilisation of multi-omic investigation a also evident, but these cases have focused more on genomics and transcriptomics [17].

The Coldest Journey Trans-Antarctic Winter Traverse (TAWT) expedition team aimed to complete a 2000-mile crossing of Antarctica in the winter (March to September 2013). This period is characterised by extreme cold, with ambient temperatures below −50 °C and three months of complete darkness. Initially, the TAWT expedition was to be undertaken from March 2013 to September 2013, with several months on either side to prepare for the expedition and subsequent uplift. The progression of the TAWT expedition was halted in June 2013, and the expedition team held firm in the interior of Antarctica. The five-man expedition team endured extreme environmental and physiological conditions for the remainder of the period in Antarctica, serving as a useful and realistic situation analogous to prolonged space travel. Here, we present what we believe is the first study to explore the multi-site response of the human microbiota (saliva and stool) and metabolome (saliva, stool, and plasma) to long-term environmental and physiological stress, which may be features of space travel.

## 2. Results and Discussion

**Figure 1:**
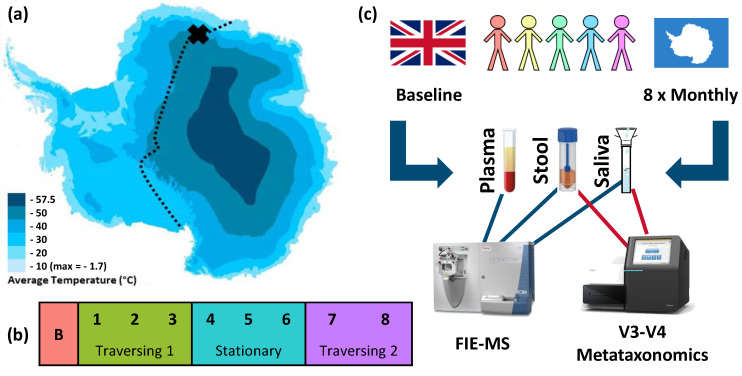
TAWT journey and experimental methodology of multi-omic analysis. (**a**) Planned route for TAWT journey with X showing the location of stationary campus after first period of traversing, adapted to show average temperatures [18]; (**b**) division of collected samples based on activity period used in statistical analysis: B = baseline; outward traversing period of months 1 to 3, stationary period of months 4 to 6, and return traversing period of months 7 and 8; and (**c**) experimental methodology of sampling from five participants with omic analysis of FIE-MS metabolic fingerprinting of plasma, stool, and saliva and metataxonomic fingerprinting of stool and saliva.

The TAWT expedition did not complete its initial intention to cross the Antarctic during the winter months of March to September. Due to the dangers posed by unexpectedly large crevasse fields, the expedition was halted during the fourth month. This was preceded by three months of traversing, where a total distance of 300 km was travelled. After three months of remaining in their stationary position, indicated in Figure 1a, the TAWT expedition undertook a further two months of traversing back to their starting position. This activity is detailed in Figure 1b and used as the framework for statistical analysis of data sets generated under the experimental workflow shown in Figure 1c. The TAWT expedition team consisted of five males, with a mean age of 37 (range 28 to 54), as detailed in Supporting Data Matrix S1, alongside individualised physiological information collected throughout the expedition.

### 2.1. Amplicon Sequencing of the V3–V4 Region of the 16S rRNA Gene Shows Differential Effect of TAWT Journey on Saliva and Stool Microbiota

**Figure 2:**
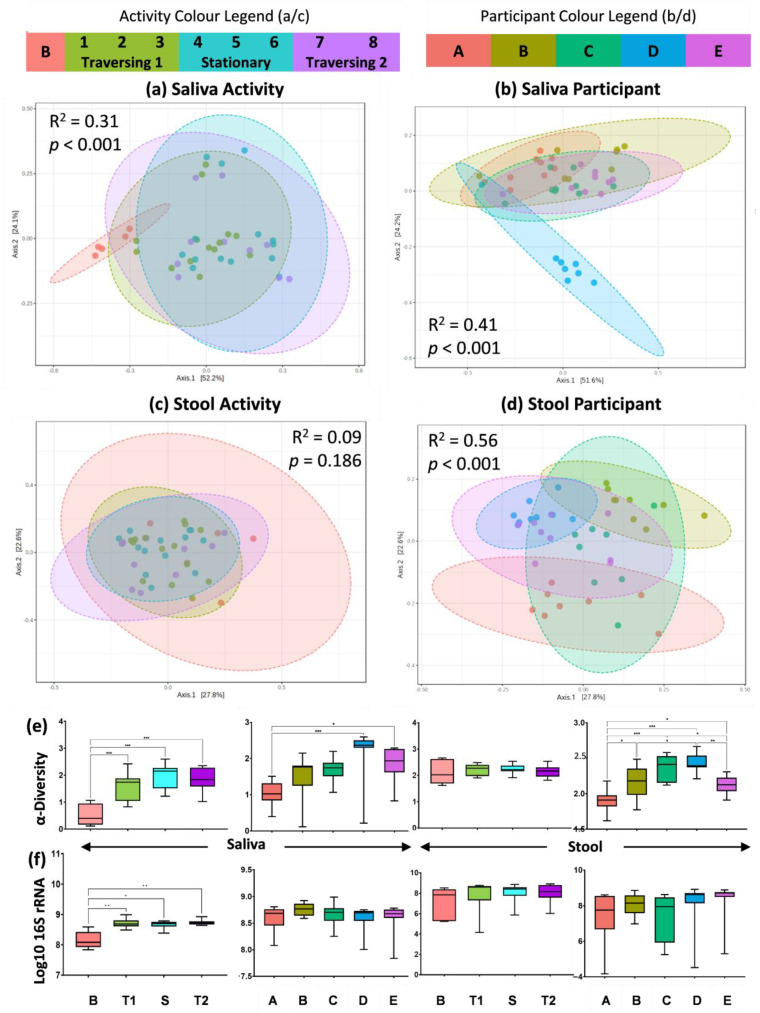
Metataxonomic fingerprinting shows spatially distinct effect of TAWT journey. (**a**–**d**) PCoA of beta diversity based on Bray–Curtis index of OTU features with statistical results from PERMONOVA analysis for saliva and stool based on activity and participant differences; (**e**) measure of alpha diversity using Shannon metric; and (**f**) measure of bacterial load based on 16S rRNA gene copy number. Statistical significance indicated by * = *p* < 0.05; ** = *p* < 0.01; and *** = *p* < 0.001.

Metataxonomic sequencing of the V3 to V4 regions of the 16S rRNA gene was used to obtain compositional data on the salivary and stool microbiota (Figure 2). The human microbiota displays spatially different responses to the physiological and environmental stressors of the TAWT expedition. For saliva, activity-related differences could be observed in beta-diversity analysis, shown in Figure 2a, between the baseline and samples collected during the expedition (R^2^ = 0.31, *p* < 0.001). It should also be noted that participant differences in the salivary microbiota were also observed (R^2^ = 0.40, *p* < 0.001), with Participant D showing maintained individual differences through the expedition, as seen in Figure 2b. Within stool microbiota metataxonomics, no significant (R^2^ = 0.09, *p* = 0.186) activity-related differences were observed, but significant (R^2^ = 0.56, *p* < 0.001) individual differences were maintained throughout the expedition. These patterns are supported by significant differences between alpha-diversity measures using the Shannon diversity metric (Figure 2e) in saliva between baselines and expedition activity periods and in both saliva and stool between at least two participants. With regard to the 16S rRNA gene copy number (Figure 2f), which has previously been suggested as a marker of immunity [19], significant (*p* < 0.001) differences were only seen between baseline and expedition saliva samples.

**Figure 3:**
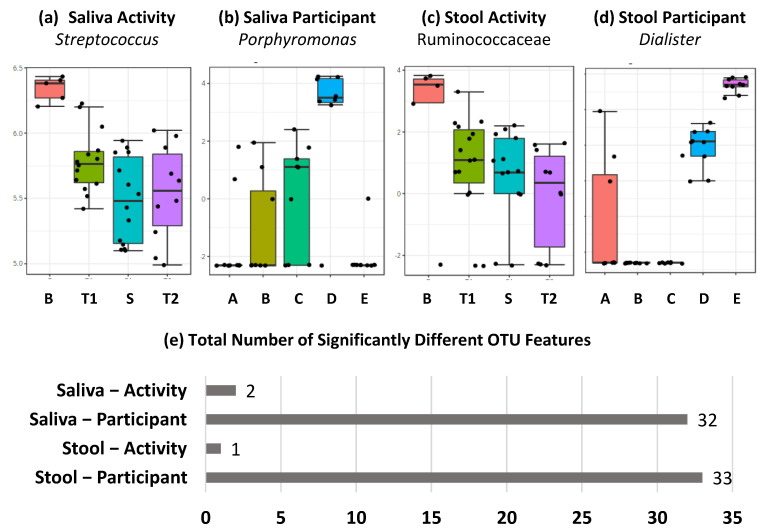
Feature level differences in metataxonomics dominated by individual differences. (**a**–**d**) Top-ranked OTU level differences based on FDR corrected *p* value across sample types and activity/participant differences labelled by lowest taxonomic classification achieved, and (**e**) total number of significantly different OTU features across sample types and activity/participant differences, with FDR-corrected *p* value below 0.05.

At the individual OTU level, seen in Figure 3, significant differences were observed across both sample (saliva and stool) and comparison (activity and participant) types. These were, however, dominated by significant differences between participants in both saliva and activity, with only *Streptoccous* and *Prevotella melaninogenica* in saliva and Ruminococcaceae family in stool significantly altered by expedition activity. As with diversity measures, these differences were seen between the baseline and samples collected during the expedition. For participant comparisons, significant differences in 32 and 33 OTUs, for saliva and stool, respectively, were observed. The lowest taxonomic identifications for these OTUs are listed in Supplementary Tables S1 and S2 for saliva and stool, respectively.

We have previously shown that the salivary microbiota is stable over a one-year period in terms of microbiota composition but there were increases in bacterial load, as measured by the 16S rRNA gene copy number, in winter months [20]. We have previously suggested that the bacterial load in saliva is indicative of immune response in athletes following periods of extreme energy expenditure [19]. Here, we observe that both bacterial load and diversity were significantly higher during the TAWT expedition when compared with baseline samples. This may be indicative of an impaired immune status that allows expansion of the ecological niche in saliva and potentially the wider oral cavity. Significant reductions in salivary IgA have been previously demonstrated in athletes after periods of intensive physical activity, which is further associated with an increased risk of upper respiratory tract infections [21]. Due to logistical limitations, analysis of immunity markers was not possible in this study. Nevertheless, these results suggest that the salivary microbiota significantly increased in terms of both bacterial load and diversity during the expedition period. As these metrics were not only higher during traversing periods, it suggests that they are not caused solely by environmental stressors and may be impacted by stresses related to isolation and reduced environmental interaction. Although the level of taxonomic identification was not sufficient to identify the presence of pathogens in the saliva, this may indicate an increased risk of infection during periods of extreme stress and/or isolation. Interestingly, acidification of saliva was also observed during the later stages of the TAWT expedition, shown in Supplementary Figure S1, which could be associated with increased pathogen load in dental caries [22]. Interestingly, a related study on the effect of isolation during the Mars-500 experiment suggested that individual taxonomic features of the salivary microbiota remained stable over the study’s duration [23].

With stool samples, no change in bacterial diversity or load was observed throughout the expedition, indicating the maintenance of prior individual differences. This is in line with observations of the Mars-500 isolation study, although reduced temporal fluctuations were observed at the OTU level [24]. The resilience of the stool microbiota is particularly noteworthy when considering the changes in dietary intake in terms of calorific content and type, such as freeze-dried meals, associated with the TAWT expedition. The one OTU that showed a significant decrease from baseline from the beginning of the expedition period was assigned to the Ruminococcaceae family. Although our taxonomic resolution prevented detailed insight, members of the Ruminococcaceae family have been associated with perceived health benefits of dietary fibre [25]. This may suggest that dietary changes, rather than environmental or physical stresses, during the TAWT expedition had a greater influence on stool microbiota composition. Stool water content, as determined by weight loss during lyophilisation, also showed significant participant differences and none associated with activity, as seen in Supplementary Figure S2. Stool consistency has previously been shown to be a major determinant of the composition of the stool microbiota, likely associated with transit time through the gastrointestinal tract [26].

### 2.2. FTIR Fingerprinting Shows Minimal Impact of Activity during TAWT Expedition

**Figure 4:**
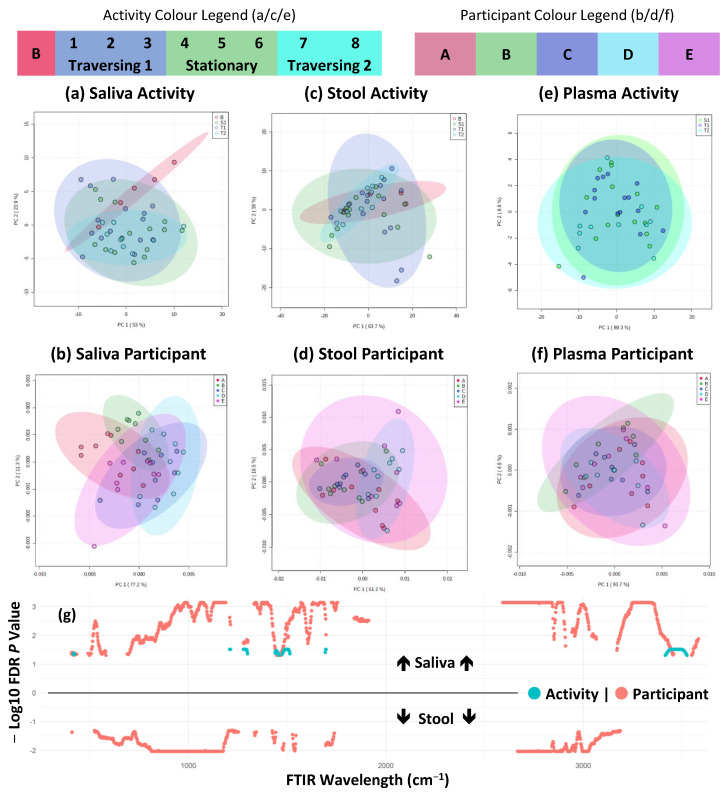
FTIR fingerprinting shows maintenance of individual difference but minor activity effects. (**a**–**f**) PCA plots of first two components across sample types and activity/participant differences; (**g**) shows significantly different (FDR corrected *p* < 0.05) FTIR fingerprint wavelengths across saliva (above line) and stool (below line) and activity/participant differences as indicated by colour shading and plotted from 400 to 4000 cm^−1^ wavelength range. No significantly different features were detected in plasma samples.

FTIR fingerprinting can provide a wide-ranging bioanalytical fingerprint of a sample. The mid-infrared wavelength (4000–600 cm^−^^1^) is divided into regions that are indicative of absorbance for key functional groups of biomolecules, including fatty acids, amides, and polysaccharides. Considering the entire spectral fingerprint, PCA modelling, seen in Figure 4a–f, showed no significant differences between activity and participants across all three analysed biofluids (saliva, stool, and plasma). At the univariate level (i.e., each cm wavelength division) of analysis, significant differences between at least two participants were observed across the absorbance spectra in both saliva and stool and between activity periods in saliva, shown in Figure 4g. Activity-related differences were shown to be abundant within the alcohol and primary amide region of 3500 to 3400 cm^−^^1^, the primary amide region of ≈1690 cm^−^^1^, and the N-O stretching functional group region of 1550 to 1500 cm^−^^1^. Although various applications of FTIR fingerprinting have shown promise in clinical diagnostics [27], one of its limitations is the lack of analytical resolution for specific biological entities. Within this study, wavelengths associated with amide functional groups appear to be the main source of statistically significant features related to the TAWT expedition. This may indicate that at the proteome/peptidome level, the TAWT expedition participants experienced a response to the physiological stress. This has been observed in plasma in response to functional overreaching exercises [28] and in the responses of mice [29] and humans [30] to space travel. Although not directly comparable, particularly with regard to the effect of zero gravity, it cannot be excluded that the TAWT expedition had an impact on participants at the proteome/peptidome level, which may have important implications for endurance and health.

Using time series analysis, a significant interaction (*p* < 0.001) was observed in FTIR fingerprinting between timepoint and participant variables in plasma samples (Supplementary Table S3). This potentially indicates a variable response between participants to the physiological stress of the TAWT expedition. This may have important biological implications, but due to the limitations of experimental design and subsequent statistical power, this aspect is not possible to explore further. Nevertheless, based on the limited significantly different features observed through other analytical methods, it is likely that the interaction effect is statistically significant but with limited biological significance.

### 2.3. Metabolite Fingerprinting Shows Maintenance of Individual Differences during TAWT Expedition

**Figure 5:**
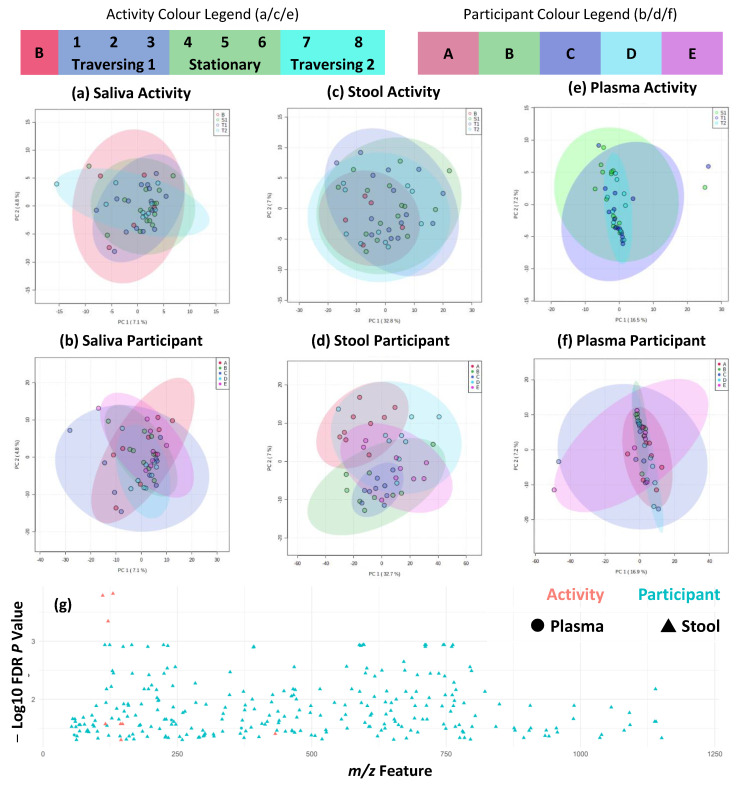
Metabolite fingerprinting shows maintenance of individual differences across traverse. (**a**–**f**) PCA plots of first two components across sample types and activity/participant differences, and (**g**) shows significantly different (FDR-corrected *p* < 0.05) metabolite fingerprint features across plasma and stool, as indicated by shape, and activity/participant differences, as indicated by colour shading and plotted from 50 to 1200 m/z range. No significantly different features were detected in saliva samples. Figure shows negative ion detection mode FIE-MS metabolite fingerprints. Equivalent figures for positive ion detection mode FIE-MS metabolite fingerprints are given in Supplementary Figure S3.

Greater insights into metabolite variation can be provided by flow-infusion electrospray mass spectrometry, and this was used to fingerprint saliva, stool, and plasma samples. This provided a snapshot of each participant’s metabolism at the time of sampling and can suggest the identification of key metabolites. For logistical reasons, baseline plasma samples were not collected from expedition participants. In line with metataxonomic results, individual differences substantially outweighed the minimal activity-associated differences across all three biofluids. Unsupervised PCA of negative ion detection mode data was completed to prevent the overfitting of models based on small participant numbers (Figure 5a–f). These indicated no large-scale shift in metabolite fingerprints associated with activity across all three biofluids, and only participant differences were observed in stool samples. At the univariate metabolite fingerprint level, as seen in Figure 5g, 282 features were significantly different between at least two participants. Of these, three were detected in plasma and 279 were detected in stool samples. In stool, a total of eight features significantly differed between at least two activity periods. Tentative identifications of metabolites are given in Supplementary Data Matrix S2. A complementary analysis of positive ion detection mode data is shown in Supplementary Figure S3 and shows similar patterns, but fewer features were significantly different between participants. Time series analysis was completed and showed no significant (*p* > 0.05) interaction between timepoint and participant variables in all three biofluids and in both ion detection mode data sets (Supplementary Table S3).

In negative ion detection mode data, six out of the eight significantly different features were not identified through a comparison of detected mass-to-charge features against the HMDB. Homovanillic acid sulphate was tentatively identified as a [M-2H]- ion and has been previously detected in human stool samples [31] and in plasma, and concentrations have been associated with levels of dopamine in the brain [32]. Homovanillic acid sulphate has not only been suggested as a marker of renal disease in adults [33] but also as a marker of bacterial biotransformation of dietary phenols [34]. It was not possible to link this with the microbiotas, as homovanillic acid sulphate metabolism is not a feature of the only bacterial taxon significantly differing in the stool, Ruminococcaceae. However, changes in the functional capacity and/or activity of the stool microbiota during the TAWT expedition would not be detected using metataxonomic sequencing. The second metabolite, tentatively identified as the [M+K-2H]- adduct of 6′-[(carboxymethyl)-C-hydroxycarbonimidoyl]-2′,3′,4,4′,5,6-hexahydroxy-[1,1′-biphenyl]-2-carboxylic acid, may have been a product of biotransformation from dietary intake. In positive ion detection mode data, four of the 12 features that significantly differed between at least two activity periods were tentatively identified: trans-3-feruloylcorosolic acid, coumesterol, acutilobin, and a pyranoflavonoid. None of these metabolites have a reported link with stress, isolation, or physical activity in the published literature. Considering the extent of individual participant differences, the minimal effect of the TAWT expedition on the detected metabolome suggests that participants were minimally impacted in terms of metabolic function at the point of host metabolism and host/microbiota interaction, considering the range of biofluids sampled in this study. Metabolomics in endurance athletes has been studied as a route to a mechanistic understanding of performance [35] and appears to be particularly associated with endurance capacity [36]. The TAWT expedition did not have a non-stressed control group and thus, direct comparisons are not experimentally possible. Temporal stability of the saliva, stool, and plasma metabolite fingerprints observed at the individual level suggests that expedition participants possess a high degree of physiological endurance. Thus, metabolomic profiles of potential participants in long-term expeditions, including space travel, may act as a useful predictor of endurance potential and act as a baseline against which perturbations may be indicative of impaired function.

### 2.4. Multi-Omic Correlation Indicates Potential for Microbiota Modulation in Extreme Stress

**Figure 6:**
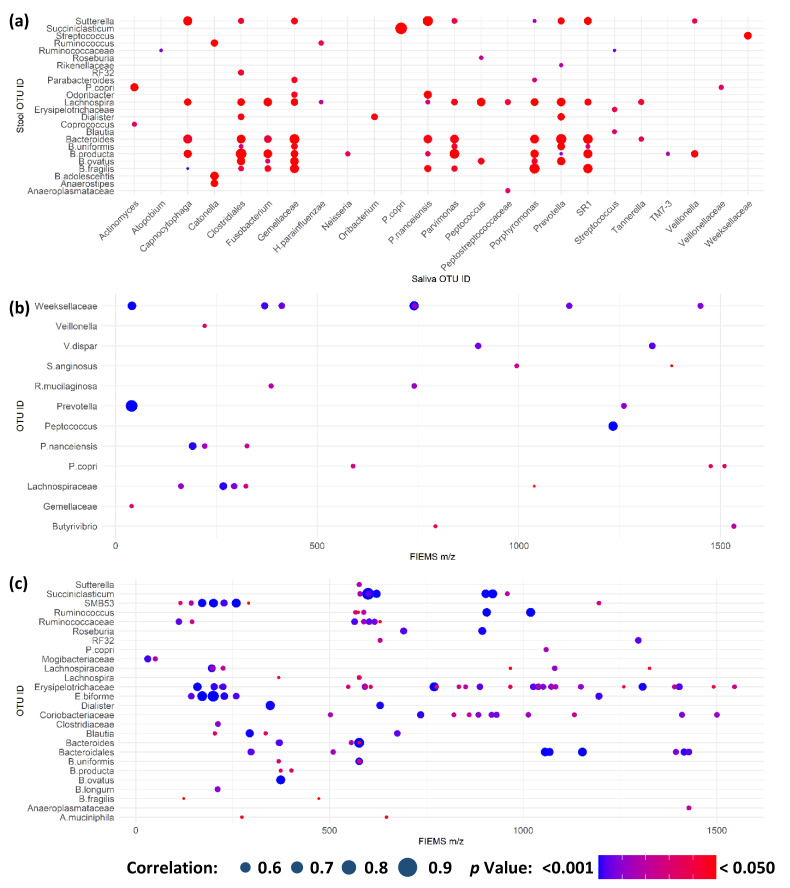
Multi-omic correlations indicate potential for microbiota modulation in extreme stress. (**a**) Significant correlations between metataxonomic features in saliva and stool samples from the same participant and sampling timepoint; (**b**) significant correlations between salivary metataxonomic and metabolite fingerprinting features; and (**c**) significant correlations between stool metataxonomic and metabolite fingerprinting features. Across all panels, only those features with at least one significant correlation are shown. Size of point is indicative of strength of correlation with only positive correlations identified in analysis. Colour of point is indicative of Bonferroni-corrected *p* value.

As a result of the combination of multi-omic analyses across saliva and stool samples, possible interactions or relationships between host and microbiota metabolism could be explored. To accomplish this, a correlation analysis between metataxonomic and metabolite fingerprints for saliva and stool biofluids was completed, shown in Figure 6. Firstly, metataxonomic feature correlations between matched timepoint/participant saliva and stool samples were completed (Figure 6a). This explored a potential link between oral and gastrointestinal microbiota as a route for the mechanistic understanding of systemic disease from changes in the oral microbiota [37]. Here, we found no correlation between any same taxon within both time- and participant-matched saliva and stool samples. We did, however, find correlations between different taxonomic groups between matched samples. It has been previously suggested that changes in either the oral cavity or gastrointestinal tract may cause systemic microbiota changes in other systems due to impaired immunological function [38]. We did not see a significant biological effect of the TAWT expedition in either saliva or stool associated with the activity period, so it can be considered unlikely to be an important factor in this analysis. A greater number of correlations of higher statistical significance were seen in both saliva and stool samples, as shown in Supplementary Figures S4 and S5, respectively. Correlations between microbiota taxa have been shown in disease states, such as periodontal disease state and severity [39]. In this study, because of technical limitations of sample numbers, it was not possible to look at whether the strength of correlation was associated with the activity period. It may be that these correlations represent multi-species communities within each ecological niche, which may be differentially affected by physiological and endurance stressors. In stool samples, many microbial taxa identified in these clusters, such as Bifidobacterium species, are associated with gut health. It may be possible to provide probiotic supplementation tailored to each baseline microbiota composition during future expeditions to maintain composition and potentially host health.

Within each sample type, metataxonomic and metabolite fingerprinting data were combined to assess if there were correlations that may be indicative of host-microbiota interaction or potential prebiotic compounds (Figure 6b,c) for saliva and stool, respectively. Positive ion detection mode correlations are shown in Supplementary Figures S6 and S7 for saliva and stool, respectively. In both ion detection modes, stool samples showed the greatest number of correlating features that are expected when considering the greater microbial and metabolite abundance and complexity within the gastrointestinal tract [40]. Of note is the large number of metabolites within the stool, which are significantly correlated with a metataxonomic OTU and significantly different between participants, as detailed in Supplementary Data Matrix S2. As participant differences were the greatest separating factor modelled in both metataxonomic and metabolomic fingerprinting analyses, this raises the potential that these features are by-products of host–microbiota interactions or dietary prebiotics that assist in modulation of the gastrointestinal microbiota [41]. Due to the observational nature of this study, it is not possible to explore this further. Further work is needed to better understand the relationship between prebiotics and probiotics in the gut and salivary microbiotas and how they could be utilised to maximise long-term health during future long-term endurance journeys.

## 3. Materials and Methods

### 3.1. Ethics Statement and Role of Funding Source

The White Mars study protocol, from which samples were received for this study, formed part of the TAWT expedition that took place from December 2012 to September 2013. The White Mars protocol was hosted by King’s College London’s Centre of Human and Aerospace Physiological Sciences, which gained the necessary ethical approval to collect samples. This component of the White Mars project received additional ethical approval from the Aberystwyth University Research Ethics Committee. Informed consent was obtained from all study participants, and all participants’ information was anonymised by the expedition doctor. The funders of this work had no input into the design or reporting of this study.

### 3.2. Participant Recruitment and Sampling

A summary of the study protocol is given in Figure 1. A total of five participants took part in the White Mars study protocol undertaken during TAWT. All sampling was completed by the expedition doctor. Stool samples were collected by depositing a small amount (≈10 g) of fresh faeces, meaning after a regular bowel movement, using a polystyrene faecal storage device (Greiner Bio-One, Frickenhausen, Germany). Saliva samples were collected by stimulating saliva production through chewing and depositing into a wide-neck 30 mL universal tube, after which ≈4 mL of sample was transferred to a smaller storage container. A baseline sample (except blood plasma) was taken from all participants prior to the start of the expedition and stored at −80 °C and analysed in parallel with the expedition samples. During the expedition, stool and saliva samples were taken at monthly intervals over an eight-month period. Stool samples were donated at a time convenient to expedition members’ daily activities, whereas saliva samples were donated by participants in the morning before any food or drink was consumed and prior to any oral hygiene regimen. No restrictions were placed on participants with regard to behaviour prior to sample donation. Baseline samples were frozen at −25 °C throughout the duration of the expedition. Expedition samples were stored at outside ambient temperatures (≈ −25 to −40 °C) in an uninsulated box until external temperatures exceeded approximately −20 °C, at which point they were stored in the expedition freezer set at −40 °C. Following the end of the expedition, samples were stored at −25 °C in freezers at the Princess Elizabeth base until transferred to the UK via ship transit in cold storage where temperatures did not exceed −25 °C. Upon arrival to the UK, samples were stored at University College London (London, UK) until transfer on dry ice to Aberystwyth University laboratories, where they were stored at −80 °C until sample processing.

### 3.3. Sample Processing, DNA Extraction, and pH Measurement

From storage, the 4 mL raw saliva samples were thawed at 4 °C and placed on a vortex mixture for 30 s to homogenise the mixture. Each saliva sample was split into two 2 mL aliquots. One aliquot was frozen at −80 °C until thawed for pH measurement and metabolite fingerprinting, and the remaining aliquot underwent centrifugation at 13,000× *g* for ten minutes at 4 °C. The resulting supernatant was removed, and the remaining pellet was also immediately frozen at −80 °C until used for DNA extraction, which was completed within seven days of receiving the sample. Blood plasma samples were thawed at 4°C and underwent centrifugation at 13,000× *g* for ten minutes at 4 °C to ensure no separation (which would be indicative of a mixed blood sample) and 2 mL transferred to a sterile 2 mL microcentrifuge tube and immediately frozen at −80 °C until thawed for pH measurement and metabolite fingerprinting. Stool samples were transferred to sterile, pre-weighed glass vials and weighed to determine wet weight. Stool samples were frozen at −20 °C and transferred to a freeze-drier set at −50 °C. Stool samples were then weighed every 24 h until they had reached a stable weight, determined as a weight reduction, over a 24-h period of less than 5%. After freeze-drying, stool samples were stored at −80 °C. For saliva samples, total genomic DNA was extracted using a FastDNA SPIN kit for Soil (MP Biomedical, Strasbourg, France) from the saliva pellet and 100 mg of freeze-dried stool as previously described [20] with an additional ethanol wash step included for stool samples. Separate DNA extraction controls were included for saliva and stool extraction batches. Measurements of plasma and saliva pH were completed in duplicate using a B-212 Twin pH meter (Horiba, Japan) after two-point calibration using a pH 7 and pH 4 buffer. For each measurement, 200 µL of sample was deposited on the sensor, ensuring full coverage of both sensor points. After stabilisation of the reading, the pH value was recorded, and the sensor was washed with ultrapure water and blotted dry.

### 3.4. 16S rRNA Gene Quantitative PCR

Standards for quantitative PCR were created through amplification of the entire 16S rRNA gene for each of the five baseline samples for stool and saliva separately using a protocol described previously [20]. After creation of standards, quantitative PCR was completed in duplicate on neat extracted DNA for each of the saliva and stool samples, as described previously [20]. Reactions were run using a C100 thermal cycler (BioRad, Hercules, CA, USA) and CFX96 optical detector (BioRad), with data captured using CFX Manager software (version 3.1, BioRad), under conditions of 95 °C for 10 min, 40 cycles of 95 °C for 15 s and 60 °C for 60 s, followed by a melt curve consisting of a temperature gradient of 60 °C to 95 °C in 0.5 °C increments, each for five seconds.

### 3.5. 16S rRNA Gene Amplicon Preparation, Sequencing and Analysis

The V3 to V4 region of the 16S rRNA gene was amplified through duplicate PCR alongside negative extraction controls, as previously described [20]. In brief, an initial PCR was completed to amplify the V3 to V4 region of the 16S rRNA gene using primers with Illumina overhang adapter sequences. After clean-up of combined PCR reactions, a second limited cycle PCR was completed to attach Illumina Nextera XT Index Primer 1 and 2 to allow multiplex sequencing. Following gel-based excision and purification, PCR products were quantified, and equimolar pools of sample libraries were sequenced, along with 20% PhiX DNA as a control for low diversity, on the Illumina MiSeq platform using MiSeq v3 reagents for a 2 × 300 bp run at the IBERS Translational Genomics Facility, Aberystwyth University. Sample reads were analysed using the QIIME 2 [42] pipeline as previously described [43]. In brief, reads were demultiplexed and truncated to 250 bp based on quality scores using FastQC [44] and underwent denoising, filtering, trimming, and chimera removal using DADA2 [45]. Taxonomic assignment of consensus operational taxonomic units (OTUs) was determined through classification against the Greengenes database (version 13.8) [46] using the open-reference vsearch at 97% sequence identify function. Resulting data matrices were analysed using MicrobiotaAnalyst [47], with samples C5 and A6 removed from the stool data set and samples D1 and B4 removed from the saliva data set due to low (<500) read number indicating failure of sequencing. OTUs were filtered based on a minimum count of 4 in 10% of samples and rarefied to minimum library size and scale using total sum scaling. The Shannon diversity measure was used for alpha-diversity analysis and principal coordinate analysis based on Bray–Curtis index was used for beta-diversity.

### 3.6. Flow Infusion Electrospray Ionisation Mass Spectrometry Metabolite Fingerprinting

For saliva and blood plasma, samples were thawed at 4 °C and 200 µL transferred to a sterile 2 mL microcentrifuge tube, to which 30 mg of <106 µM acetone-washed glass beads (Sigma-Aldrich) was added. To this, 1520 µL of HPLC-grade methanol and chloroform (4:1 *v*/*v*) was added. Freeze-dried stool samples were reconstituted at a concentration of 100 mg/mL in HPLC-grade water, methanol, and chloroform (2:5:2 *v*/*v*), and 30 mg of <106 µM acetone-washed glass beads was added. Samples were homogenised through vortex mixing for 5 s and then milling for 30 s at 5 Hz, after which they were shaken for 20 min at 4 °C and then stored at −20 °C for 20 min to precipitate protein. Samples then underwent centrifugation at 13,000× *g* for 6 min at 4 °C and the supernatant was transferred to a clean 2 mL microcentrifuge tube. Stool samples were diluted by 50% in the water, methanol, and chloroform mixture used in extraction in a clean 2 mL microcentrifuge tube. Samples were analysed using an LTQ linear ion trap (ThermoFisher Scientific, Waltham, MA, USA) as previously described [48], with 20 µL of sample injected into a water–methanol (7:3 *v*/*v*) solvent mix running at a flow rate of 60 µL/min. Data matrices were analysed using MetaboAnalyst [49] using total ion count normalisation, log transformation, and Pareto scaling. Tentative metabolite identifications were assigned using accurate mass matches to the Human Metabolome Database [50] with a 10 ppm cut-off.

### 3.7. Fourier Transformed Infrared Spectroscopy Fingerprinting

Unprocessed saliva and plasma and re-suspended stool samples in the solvent mix used for FIE-MS analysis were thawed at 4 °C from storage at −80 °C. Within each group, samples were randomised and 5 µL spotted onto duplicate 96 well silica transmission plates and dried at 55 °C for 30 min to remove residual solvent and water. Each plate was analysed using an Equinox 55 instrument (Bruker, Coventry, UK) with an attached HTS-XT microplate reader (Bruker) in transmission mode. The background level for each plate was determined by a blank well, and average values from over 32 readings were taken for each detected wavelength over the 4000–600 cm^−1^ region. The average value for each wavenumber for each duplicate reading was calculated and subjected to background subtraction and total intensity count normalisation. Data matrices were analysed using MetaboAnalyst [49] using log transformation and Pareto scaling.

### 3.8. Statistical and Data Analysis

Univariate data sets (16S rRNA qPCR and pH) were analysed using Prism 8 software (GraphPad Software, San Diego, CA, USA) after means of duplicate readings and using one-way ANOVA with *post-hoc* Tukey’s test corrected for multiple comparisons. Univariate analysis completed in MetaboAnalyst was exported and formatted on a mass-to-charge/wavelength axis for visualisation in the R environment. A false discovery rate corrected *p* value threshold of less than 0.05 was used for the identification of significant univariate features in both MicrobiotaAnalyst and MetaboAnalyst analyses. Multi-omic correlations were completed between normalised data sets in the R environment using Bonferroni correction for multiple hypothesis correction.

## 4. Conclusions

The effect of the TAWT expedition on the human microbiota and metabolome has been shown to be spatially different across bodily sites. Significant activity-associated changes in terms of both bacterial diversity and load were seen in saliva but not in stool, and participant differences in metabolite fingerprints persisted across all three sample types. Although the TAWT expedition was associated with considerable environmental and physiological stress, the observations noted in this work may also be associated with changes in the day-to-day conditions of the participants, in terms of close quarters and small group living. This may be of particular interest when considering the living conditions for long-term space travel. Further work beyond simulated exercises is needed to understand the overall interaction of these biological systems and how they impact host health and fitness and should form part of future studies in tracking responses to long-term space travel or their terrestrial proxies. It is further justified by this work to expand analysis beyond microbiota profiling to include broader measures of system function, including metabolomic profiling.

## Data Availability

Raw sequence reads have been deposited at the European Nucleotide Archive under primary accession number PRJEB9027 and secondary accession number ERP010081. Processed data matrices for FIE-MS and FTIR fingerprinting data sets are available as csv files as detailed in the Supporting Information for this manuscript.

## Supplementary Materials

The following supporting information can be downloaded at: https://www.mdpi.com/article/10.3390/ijms24054850/s1.

## References

[1] Kinross J.M., Darzi A.W., Nicholson J.K. (2011). Gut microbiome-host interactions in health and disease. Genome Med..

[2] Li X., Kan E.M., Lu J., Cao Y., Wong R.K., Keshavarzian A., Wilder-Smith C.H. (2013). Combat-training increases intestinal permeability, immune activation and gastrointestinal symptoms in soldiers. Aliment. Pharmacol. Ther..

[3] Groeneweg F.L., Karst H., de Kloet R., Joels M. (2011). Rapid non-genomic effects of corticosteroids and their role in the central stress response. J. Endocrinol..

[4] Dinan T.G., Cryan J.F. (2012). Regulation of the stress response by the gut microbiota: Implications for psychoneuroendocrinology. Psychoneuroendocrinology.

[5] Gareau M.G., Jury J., MacQueen G., Sherman P.M., Perdue M.H. (2007). Probiotic treatment of rat pups normalises corticosterone release and ameliorates colonic dysfunction in-duced by maternal separation. Gut.

[6] Heijtz R.D., Wang S., Anuar F., Qian Y., Björkholm B., Samuelsson A., Hibberd M.L., Forssberg H., Pettersson S. (2011). Normal gut microbiota modulates brain development and behavior. Proc. Natl. Acad. Sci. USA.

[7] Ochoa-Repáraz J., Mielcarz D.W., Haque S.B., Kasper L.H. (2010). Gut, bugs, and brain: Role of commensal bacteria in the control of central nervous system disease. Ann. Neurol..

[8] Wang Y., Kasper L.H. (2013). The role of microbiome in central nervous system disorders. Brain Behav. Immun..

[9] Messaoudi M., Lalonde R., Violle N., Javelot H., Desor D., Nejdi A., Bisson J.F., Rougeot C., Pichelin M., Cazaubiel M. (2011). Assessment of psychotropic-like properties of a probiotic formulation (Lactobacillus helveticus R0052 and Bifidobacterium longum R0175) in rats and human subjects. Br. J. Nutr..

[10] Saei A.A., Barzegari A. (2012). The microbiome: The forgotten organ of the astronaut’s body—Probiotics beyond terrestrial limits. Future Microbiol..

[11] Singh N.K., Wood J.M., Karouia F., Venkateswaran K. (2018). Succession and persistence of microbial communities and antimicrobial resistance genes associated with International Space Station environmental surfaces. Microbiome.

[12] Checinska Sielaff A., Urbaniak C., Mohan G.B.M., Stepanov V.G., Tran Q., Wood J.M., Minich J., McDonald D., Mayer T., Knight R. (2019). Characterization of the total and viable bacterial and fungal communities associated with the International Space Station surfaces. Microbiome.

[13] da Silveira W.A., Fazelinia H., Rosenthal S.B., Laiakis E.C., Kim M.S., Meydan C., Kidane Y., Rathi K.S., Smith S.M., Stear B. (2020). Comprehensive multi-omics analysis reveals mitochondrial stress as a central biological hub for space-flight impact. Cell.

[14] Mardanov A.V., Babykin M.M., Beletsky A.V., Grigoriev A.I., Zinchenko V., Kadnikov V.V., Kirpichnikov M.P., Mazur A.M., Nedoluzhko A., Novikova N.D. (2013). Metagenomic Analysis of the Dynamic Changes in the Gut Microbiome of the Participants of the MARS-500 Experiment, Simulating Long Term Space Flight. Acta Nat..

[15] Douglas G., Voorhies A. (2017). Evidence based selection of probiotic strains to promote astronaut health or alleviate symptoms of illness on long duration spaceflight missions. Benef. Microbes.

[16] Cahill T., Hardiman G. (2020). Nutritional Challenges and Countermeasures for Space Travel.

[17] Beheshti A., Chakravarty K., Fogle H., Fazelinia H., da Silveira W.A., Boyko V., Polo S.-H.L., Saravia-Butler A.M., Hardiman G., Taylor D. (2019). Multi-omics analysis of multiple missions to space reveal a theme of lipid dysregulation in mouse liver. Sci. Rep..

[18] Morgan F., Barker G., Briggs C., Price R., Keys H., Zealand A.N. (2007). Environmental Domains of Antarctica Version 2.0 Final Report.

[19] Jones A.W., Cameron S., Thatcher R., Beecroft M.S., Mur L., Davison G. (2014). Effects of bovine colostrum supplementation on upper respiratory illness in active males. Brain Behav. Immun..

[20] Cameron S., Huws S., Hegarty M.J., Smith D., Mur L.A.J. (2015). The human salivary microbiome exhibits temporal stability in bacterial diversity. FEMS Microbiol. Ecol..

[21] Trochimiak T., Hübner-Woźniak E. (2012). Effect of exercise on the level of immunoglobulin A in saliva. Biol. Sport.

[22] Zhou J., Jiang N., Wang Z., Li L., Zhang J., Ma R., Nie H., Li Z. (2017). Influences of pH and Iron Concentration on the Salivary Microbiome in Individual Humans with and without Caries. Appl. Environ. Microbiol..

[23] Bacci G., Mengoni A., Emiliani G., Chiellini C., Cipriani E.G., Bianconi G., Canganella F., Fani R. (2021). Defining the resilience of the human salivary microbiota by a 520-day longitudinal study in a confined envi-ronment: The Mars500 mission. Microbiome.

[24] Turroni S., Rampelli S., Biagi E., Consolandi C., Severgnini M., Peano C., Quercia S., Soverini M., Carbonero F.G., Bianconi G. (2017). Temporal dynamics of the gut microbiota in people sharing a confined environment, a 520-day ground-based space simulation, MARS500. Microbiome.

[25] Cui J., Lian Y., Zhao C., Du H., Han Y., Gao W., Xiao H., Zheng J. (2019). Dietary fibers from fruits and vegetables and their health benefits via modulation of gut microbiota. Compr. Rev. Food Sci. Food Saf..

[26] Vandeputte D., Falony G., Vieira-Silva S., Tito R.Y., Joossens M., Raes J. (2016). Stool consistency is strongly associated with gut microbiota richness and composition, enterotypes and bacterial growth rates. Gut.

[27] Mitchell A.L., Gajjar K.B., Theophilou G., Martin F.L., Martin-Hirsch P.L. (2014). Vibrational spectroscopy of biofluids for disease screening or diagnosis: Translation from the laboratory to a clinical setting. J. Biophotonics.

[28] Nieman D.C., Groen A., Pugachev A., Vacca G. (2018). Detection of Functional Overreaching in Endurance Athletes Using Proteomics. Proteomes.

[29] Tascher G., Brioche T., Maes P., Chopard A., O’Gorman D., Gauquelin-Koch G., Blanc S., Bertile F. (2017). Proteome-wide Adaptations of Mouse Skeletal Muscles during a Full Month in Space. J. Proteome Res..

[30] Brzhozovskiy A.G., Kononikhin A.S., Pastushkova L.C., Kashirina D.N., Indeykina M.I., Popov I.A., Custaud M.-A., Larina I.M., Nikolaev E.N. (2019). The Effects of Spaceflight Factors on the Human Plasma Proteome, Including Both Real Space Missions and Ground-Based Experiments. Int. J. Mol. Sci..

[31] Gao X., Pujos-Guillot E., Martin J.-F., Galan P., Juste C., Jia W., Sebedio J.-L. (2009). Metabolite analysis of human fecal water by gas chromatography/mass spectrometry with ethyl chloroformate derivatization. Anal. Biochem..

[32] Sternberg D.E., Heninger G.R., Both R.H. (1983). Plasma homovanillic acid as an index of brain dopamine metabolism: Enhancement with debrisoquin. Life Sci..

[33] Barrios C., Spector T.D., Menni C. (2016). Blood, urine and faecal metabolite profiles in the study of adult renal disease. Arch. Biochem. Biophys..

[34] Dall’Asta M., Calani L., Tedeschi M., Jechiu L., Brighenti F., Del Rio D. (2012). Identification of microbial metabolites derived from in vitro fecal fermentation of different polyphenolic food sources. Nutrition.

[35] Heaney L.M., Deighton K., Suzuki T. (2017). Non-targeted metabolomics in sport and exercise science. J. Sports Sci..

[36] San-Millán I., Stefanoni D., Martinez J.L., Hansen K.C., D’Alessandro A., Nemkov T. (2020). Metabolomics of Endurance Capacity in World Tour Professional Cyclists. Front. Physiol..

[37] Ahn J., Chen C.Y., Hayes R.B. (2012). Oral microbiome and oral and gastrointestinal cancer risk. Cancer Causes Control.

[38] Olsen I., Yamazaki K. (2019). Can oral bacteria affect the microbiome of the gut?. J. Oral Microbiol..

[39] Kageyama S., Takeshita T., Asakawa M., Shibata Y., Takeuchi K., Yamanaka W., Yamashita Y. (2017). Relative abundance of total subgingival plaque-specific bacteria in salivary microbiota reflects the overall periodontal condition in patients with periodontitis. PLoS ONE.

[40] Weng Y.J., Gan H.Y., Li X., Huang Y., Li Z.C., Deng H.M., Chen S.Z., Zhou Y., Wang L.S., Han Y.P. (2019). Correlation of diet, microbiota and metabolite networks in inflammatory bowel disease. J. Dig. Dis..

[41] Tuohy K.M., Probert H.M., Smejkal C.W., Gibson G.R. (2003). Using probiotics and prebiotics to improve gut health. Drug Discov. Today.

[42] Bolyen E., Rideout J.R., Dillon M.R., Bokulich N.A., Abnet C., Al-Ghalith G.A., Caporaso J.G. (2018). QIIME 2: Reproducible, interactive, scalable, and extensible microbiome data science. PeerJ.

[43] Shenker N.S., Perdones-Montero A., Burke A., Stickland S., McDonald J.A., Alexander-Hardiman K., Flanagan J., Takats Z., Cameron S.J. (2020). Metabolomic and Metataxonomic Fingerprinting of Human Milk Suggests Compositional Stability over a Natural Term of Breastfeeding to 24 Months. Nutrients.

[44] Schmieder R., Edwards R. (2011). Quality control and preprocessing of metagenomic datasets. Bioinformatics.

[45] Callahan B.J., Mcmurdie P.J., Rosen M.J., Han A.W., Johnson A.J.A., Holmes S.P. (2016). DADA_2_: High-resolution sample inference from Illumina amplicon data. Nat. Methods.

[46] DeSantis T.Z., Hugenholtz P., Larsen N., Rojas M., Brodie E.L., Keller K., Huber T., Dalevi D., Hu P., Andersen G.L. (2006). Greengenes, a Chimera-Checked 16S rRNA Gene Database and Workbench Compatible with ARB. Appl. Environ. Microbiol..

[47] Dhariwal A., Chong J., Habib S., King I.L., Agellon L.B., Xia J. (2017). MicrobiomeAnalyst: A web-based tool for comprehensive statistical, visual and meta-analysis of microbiome data. Nucleic Acids Res..

[48] Cameron S.J., Lewis K.E., Beckmann M., Allison G.G., Ghosal R., Lewis P.D., Mur L.A. (2016). The metabolomic detection of lung cancer biomarkers in sputum. Lung Cancer.

[49] Xia J., Sinelnikov I.V., Han B., Wishart D.S. (2015). MetaboAnalyst 3.0—Making metabolomics more meaningful. Nucleic Acids Res..

[50] Wishart D.S., Jewison T., Guo A.C., Wilson M., Knox C., Liu Y., Djoumbou Y., Mandal R., Aziat F., Dong E. (2013). HMDB 3.0: The Human Metabolome Database in 2013. Nucleic Acids Res..

