# Supplementary material for: Participants in the Trans-Antarctic Winter Traverse Expedition Showed Increased Bacterial Load and Diversity in Saliva but Maintained Individual Differences within Stool Microbiota and Across Metabolite Fingerprints
Source: Int J Mol Sci. 2023 Mar 2;24(5):4850. doi: 10.3390/ijms24054850 (PMC10002533; doi:10.3390/ijms24054850)
Supplement: Supplementary file 1 [file ijms-24-04850-s001.zip › tawt_esi_v1.pdf]

ELECTRONIC SUPPORTING INFORMATION

Participants in the Trans-Antarctic Winter Traverse Expedition Showed Increased Bacterial Load and Diversity in Saliva but Maintained Individual Differences within Stool Microbiota and Across Metabolite Fingerprints

Simon J. S. Cameron, Arwyn Edwards, Robert J. Lambert, Mike Stroud, Luis A. J. Mur.

CONTENTS

S1 Table S1 - Taxonomic Identification of Significant Metataxonomic Features from Saliva Samples

S2 Table S2 - Taxonomic Identification of Significant Metataxonomic Features from Stool Samples

S3 Table S3 - Time Series Analysis of FIE-MS and FTIR Metabolite Fingerprinting

S4 Figure S1 - Salivary and Plasma pH Across Activity and Participant Differences

S5 Figure S2 - Water Content of Stool Shows Participant Not Activity Differences

S6 Figure S3 - Positive Ion Detection Mode FIE-MS Metabolite Fingerprinting

S7 Figure S4 - Metataxonomic Correlations within Salivary Samples

S8 Figure S5 - Metataxonomic Correlations within Stool Samples

S9 Figure S6 - Metataxonomic Correlations with Positive Ion FIE-MS in Salivary Samples

S10 Figure S7 - Metataxonomic Correlations with Positive Ion FIE-MS in Stool Samples

S11 Supporting Data Matrix 1 - Individual Participant Metadata Across TAWT Period

S12 Supporting Data Matrix 2 - Tentative Database Matches for Metabolite Fingerprints

S13 Supporting Data Files – FTIR and FIE-MS Metabolite Fingerprinting Data Sets

Table S1. Taxonomic Identification of Significant Metataxonomic Features from Saliva Samples

Taxonomic identifications to the lowest level possible based on QIIME processing and comparison to the Greengenes database for significant metataxonomic features from saliva identified through MicrobiomeAnalyst analysis for activity and participant differences.

OTU Feature	FDR Value	Lowest Taxon ID		
**Saliva Activity**				
OTU028	5.06E-06	Genus	*Streptococcus*	
OTU012	0.00788	Species	*Prevotella*	*melaninogenica*
**Saliva Participants**				
OTU006	5.96E-08	Genus	*Porphyromonas*	
OTU021	5.96E-08	Family	Gemellaceae	
OTU030	5.96E-08	Order	Clostridiales	
OTU065	7.93E-08	Phylum	SR1	
OTU050	5.26E-07	Genus	*Parvimonas*	
OTU053	5.26E-07	Genus	*Fusobacterium*	
OTU013	1.76E-06	Species	*Prevotella*	*nanceiensis*
OTU016	4.55E-06	Genus	*Capnocytophaga*	
OTU032	6.25E-06	Family	Lachnospiraceae	
OTU055	7.20E-05	Genus	*Leptotrichia*	
OTU014	0.000406	Genus	*Prevotella*	
OTU036	0.000631	Genus	*Oribacterium*	
OTU009	0.000783	Genus	*Prevotella*	
OTU010	0.001551	Genus	*Prevotella*	
OTU045	0.00235	Genus	*Veillonella*	
OTU067	0.002867	Class	TM7-3	
OTU047	0.002916	Species	*Veillonella*	*dispar*
OTU020	0.007273	Family	Gemellaceae	
OTU041	0.007273	Family	Veillonellaceae	
OTU058	0.007446	Genus	*Neisseria*	
OTU031	0.007635	Order	Clostridiales	
OTU012	0.011791	Species	*Prevotella*	*melaninogenica*
OTU022	0.012925	Order	Lactobacillales	
OTU001	0.012925	Genus	*Actinomyces*	
OTU004	0.015672	Genus	*Atopobium*	
OTU038	0.020695	Genus	*Peptococcus*	
OTU061	0.028259	Genus	*Haemophilus*	
OTU048	0.031987	Family	Mogibacteriaceae	
OTU052	0.032108	Species	*Bulleidia*	*moorei*
OTU028	0.036996	Genus	*Streptococcus*	
OTU039	0.037449	Family	Peptostreptococcaceae	
OTU034	0.049772	Genus	*Catonella*	

Table S2. Taxonomic Identification of Significant Metataxonomic Features from Stool Samples

Taxonomic identifications to the lowest level possible based on QIIME processing and comparison to the Greengenes database for significant metataxonomic features from saliva identified through MicrobiomeAnalyst analysis for activity and participant differences.

OTU Feature	FDR Value	Lowest Taxon ID		
**Stool Activity**				
OTU055	3.59E-05	Family	Ruminococcaceae	
**Stool Participant**				
OTU061	3.04E-18	Genus	*Dialister*	
OTU056	1.62E-12	Family	Ruminococcaceae	
OTU043	3.32E-08	Species	*Blautia*	*producta*
OTU042	4.10E-08	Genus	*Blautia*	
OTU039	7.77E-08	Family	Lachnospiraceae	
OTU059	1.52E-07	Genus	*Ruminococcus*	
OTU010	5.53E-07	Genus	*Bacteroides*	
OTU011	2.31E-06	Genus	*Bacteroides*	
OTU068	4.43E-06	Family	Erysipelotrichaceae	
OTU016	5.56E-05	Genus	*Parabacteroides*	
OTU049	9.50E-05	Genus	*Lachnospira*	
OTU037	0.000245	Genus	SMB53	
OTU057	0.00043	Species	*Faecalibacterium*	*prausnitzii*
OTU014	0.000449	Species	*Bacteroides*	*ovatus*
OTU078	0.000449	Family	Anaeroplasmataceae	
OTU074	0.000757	Genus	*Sutterella*	
OTU060	0.001129	Genus	*Ruminococcus*	
OTU013	0.001143	Species	*Bacteroides*	*fragilis*
OTU020	0.006354	Species	*Prevotella*	*copri*
OTU015	0.008499	Species	*Bacteroides*	*uniformis*
OTU050	0.008499	Genus	*Roseburia*	
OTU044	0.009105	Genus	***Coprococcus***	
OTU031	0.01001	Order	Clostridiales	
OTU032	0.010225	Order	Clostridiales	
OTU003	0.019339	Species	*Bifidobacterium*	*adolescentis*
OTU041	0.019661	Genus	*Blautia*	
OTU035	0.019661	Family	Clostridiaceae	
OTU045	0.022088	Genus	*Coprococcus*	
OTU025	0.030041	Genus	*Odoribacter*	
OTU040	0.030041	Genus	*Anaerostipes*	
OTU051	0.032061	Genus	*Ruminococcus*	
OTU038	0.036159	Family	Lachnospiraceae	
OTU070	0.045024	Species	*Eubacterium*	*biforme*

Table S3. Time Series Analysis of FIE-MS and FTIR Metabolite Fingerprinting

Time series analysis using MetaboAnalyst two-way ANOVA pipeline for three metabolite fingerprinting modalities across all three biofluid sample types. *P* values given for time, participant, and interaction effects.

Analysis Method	Biofluid	Time *P* Value	Participant *P* Value	Interaction *P* Value
FIEMS Negative	Saliva	0.75	0.25	0.80
	Plasma	1.00	0.15	0.75
	Stool	1.00	< 0.05	0.65
FIEMS Positive	Saliva	0.35	0.10	0.45
	Plasma	0.80	0.25	0.20
	Stool	1.00	< 0.05	0.70
FTIR	Saliva	0.80	0.25	0.55
	Plasma	0.60	0.95	< 0.001
	Stool	0.85	< 0.001	0.25

Figure S1. Salivary and Plasma pH Across Activity and Participant Differences

Salivary and plasma pH differences across activity periods and individual participants. pH readings taken to one decimal place on raw, untreated samples. Statistical significance determined by FDR corrected one-way ANOVA and indicated by * = $P < 0.05$; ** = $P < 0.01$; and *** = $P < 0.001$.

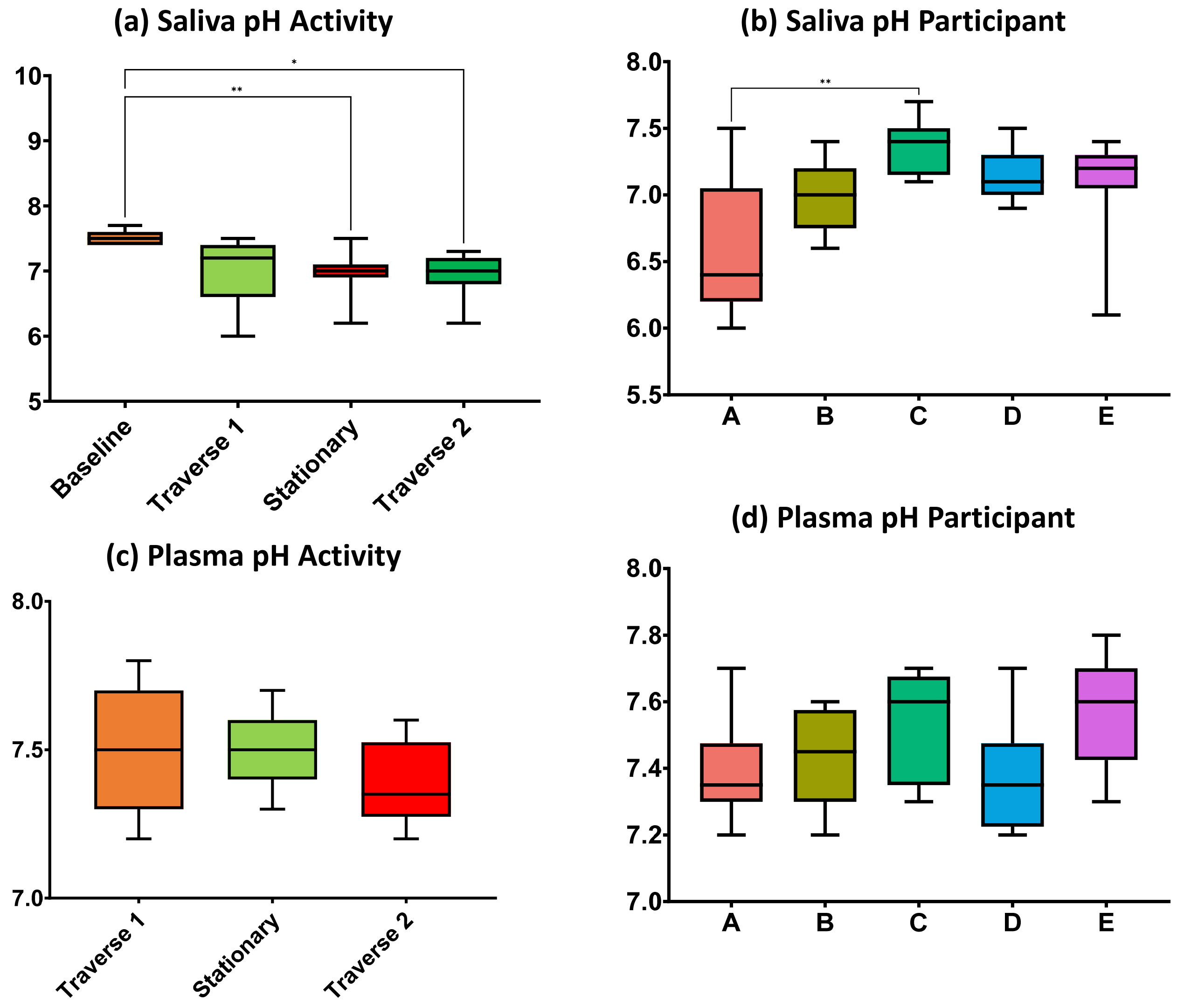

Figure S2. Water Content of Stool Shows Participant Not Activity Differences

Water content of stool samples as determined by weight loss after freeze drying process across activity periods and individual participants. Statistical significance determined by FDR corrected one-way ANOVA and indicated by * = $P < 0.05$; ** = $P < 0.01$; and *** = $P < 0.001$.

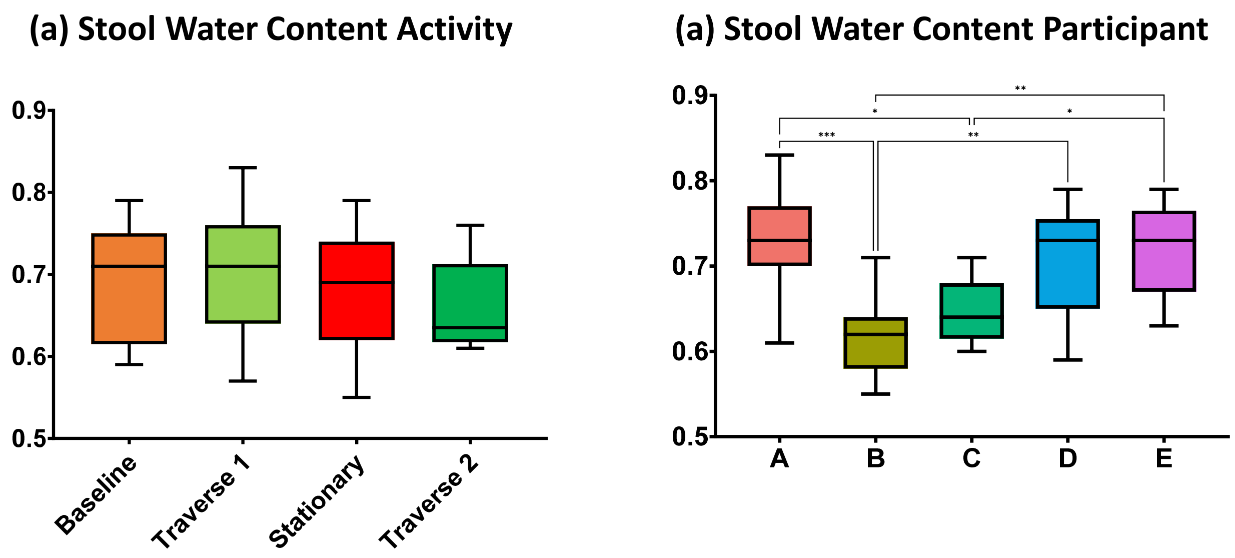

Figure S3. Positive Ion Detection Mode FIE-MS Metabolite Fingerprinting

(a-f) PCA plots of first two components across sample types and activity/participant differences; and (g) shows significantly different (FDR corrected *P* <0.05) metabolite fingerprint features in stool for activity/participant differences as indicated by colour shading and plotted from 50 to 1200 *m/z* range. No significantly different features were detected in saliva nor plasma samples.

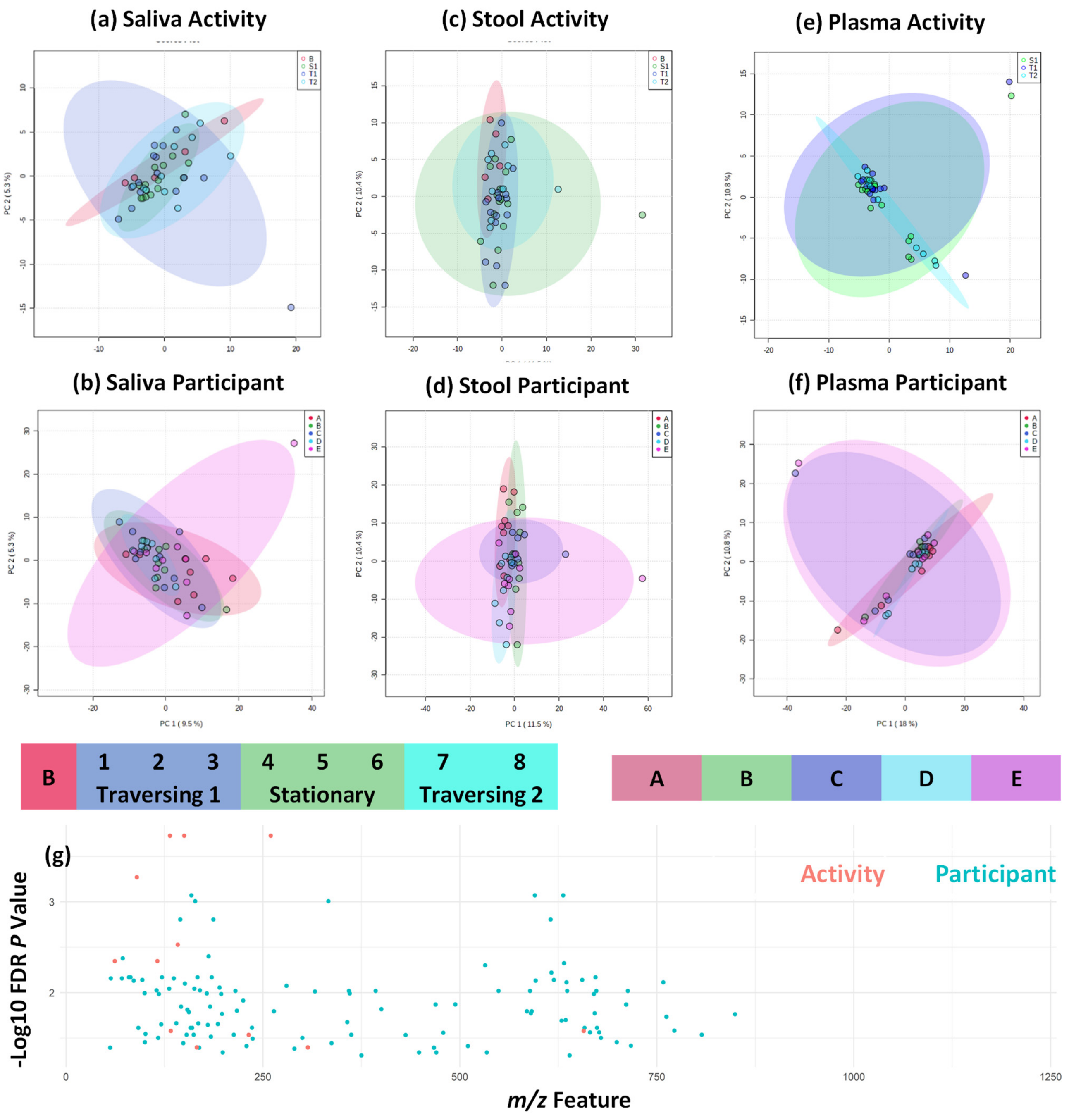

Figure S4. Metataxonomic Correlations within Salivary Samples

OTU level correlations within saliva samples shown to the lowest level of taxonomic classification. Only those taxons with at least one significant correlation ($P < 0.05$) other than with itself after Bonferroni multiple testing correction are displayed. Within these taxons, non-significant correlations ($P > 0.05$) are not shown. The size and colour of circles indicates type (blue = positive and red = negative) and strength of correlation as indicated by heat legend.

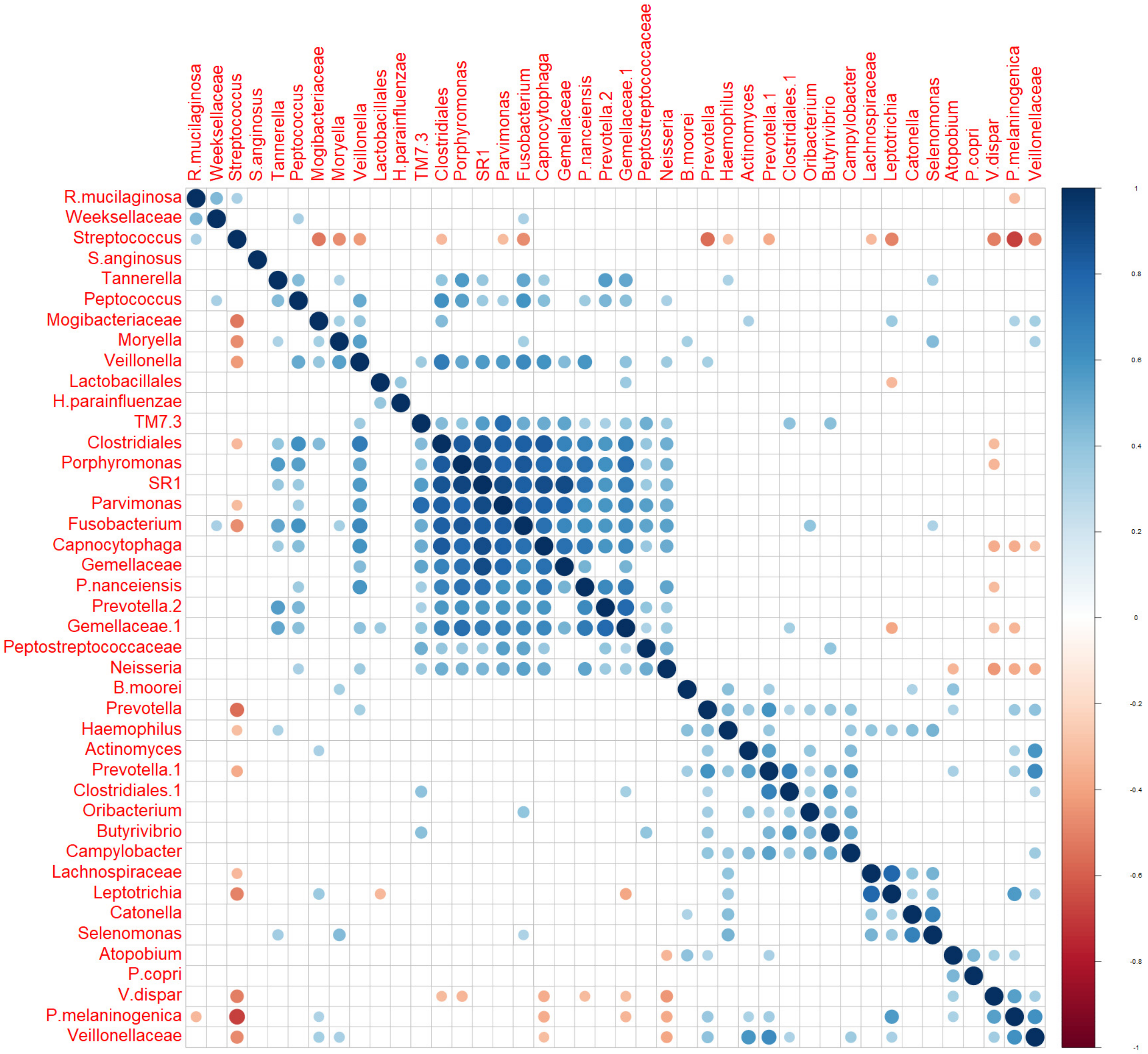

Figure S5. Metataxonomic Correlations within Stool Samples

OTU level correlations within stool samples shown to the lowest level of taxonomic classification. Only those taxons with at least one significant correlation ($P < 0.05$) other than with itself after Bonferroni multiple testing correction are displayed. Within these taxons, non-significant correlations ($P > 0.05$) are not shown. The size and colour of circles indicates type (blue = positive and red = negative) and strength of correlation as indicated by heat legend.

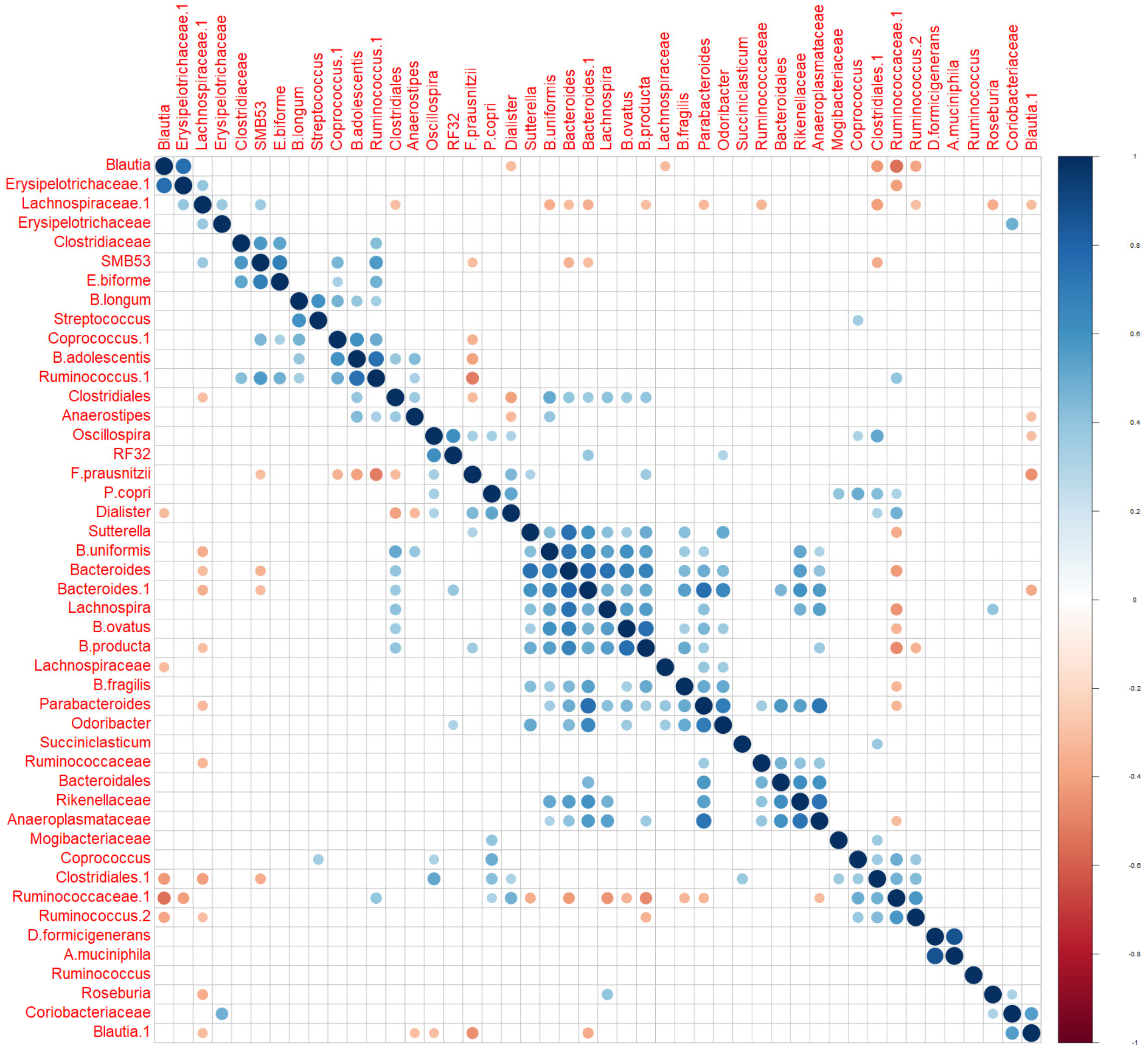

Figure S6. Metataxonomic Correlations with Positive Ion FIE-MS in Salivary Samples

Significant correlations between metataxonomic and positive ion FIE-MS metabolite fingerprint features in salivary samples. Only taxons with at least one significant correlation are shown. Correlations are plotted with size of point indicative of strength of correlation and colour of point indicative of Bonferroni corrected *P* value as shown by accompanying legend.

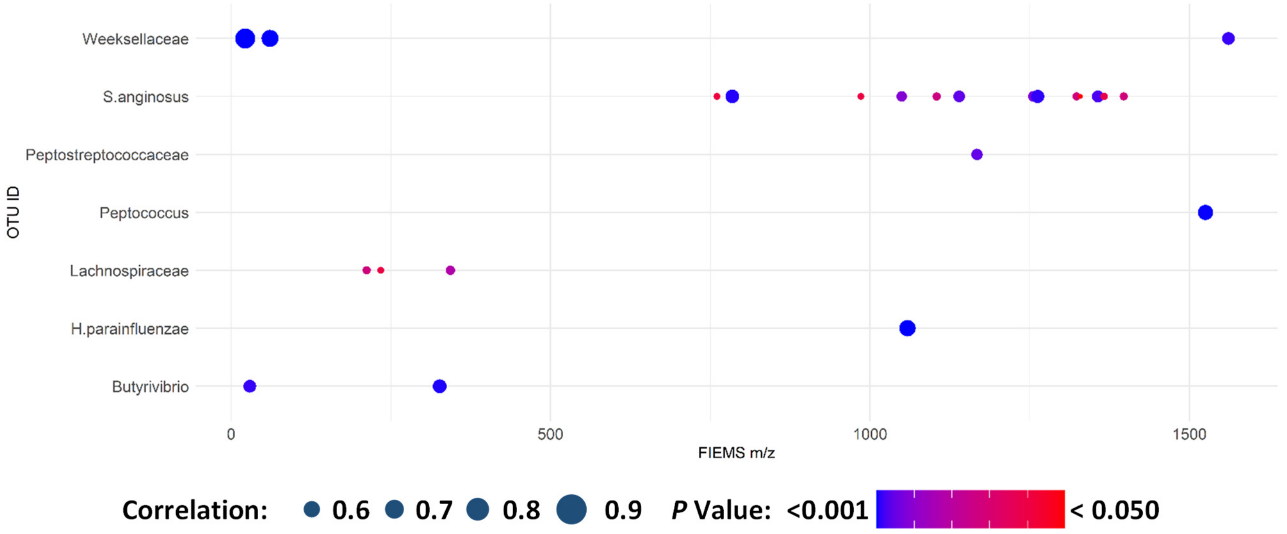

Figure S7. Metataxonomic Correlations with Positive Ion FIE-MS in Stool Samples

Significant correlations between metataxonomic and positive ion FIE-MS metabolite fingerprint features in stool samples. Only taxons with at least one significant correlation are shown. Correlations are plotted with size of point indicative of strength of correlation and colour of point indicative of Bonferroni corrected *P* value as shown by accompanying legend.

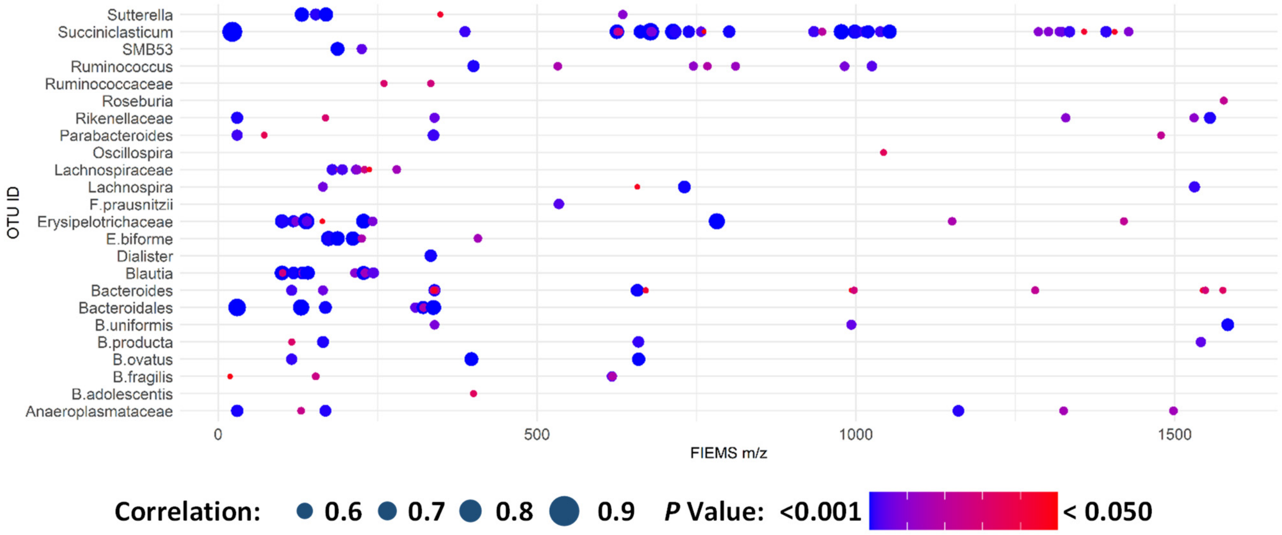

Supporting Data Matrix 1 – Individual Participant Metadata Across TAWT Period

This supplementary data matrix is provided an additional Microsoft Excel (.xlsx) file. Each tab of the spreadsheet gives information on each participant (A, B, C, D, and E) across a range of physiological parameters for each month of the TAWT period. Measurements were taken by the expedition medic using established methodologies. Provided in ZIP archive file with Supporting Data Matrix 2 and Supporting Data Files.

Supporting Data Matrix 2 – Tentative Database Matches for Metabolite Fingerprints

This supplementary data matrix is provided as an additional Microsoft Excel (.xlsx) file. Each tab of the spreadsheet gives information on the tentative database matches for metabolite fingerprint features for either negative ion or positive ion detection mode FIE-MS analysis. Only those features which have a significant result with either activity or individual participant differences or significant correlation with a metataxonomic feature are shown. Tentative database matches are given against the Human Metabolome Database with the following details recorded and as detailed in the Materials and Method section of the main manuscript. Provided in ZIP archive file with Supporting Data Matrix 1 and Supporting Data Files.

**Data Column**	**Description**
m/z	Mass-to-charge ratio of metabolite fingerprint feature
Biofluid	Biofluid (plasma, saliva, stool) feature identified in
Source	Analysis source for identification of significant difference or correlation
Count	Number of tentative database matches with the same ppm values
Name	Standardised chemical name of tentative database match
Formula	Elemental formula of tentative database match
Monoisotopic Mass	Monoisotopic mass of tentative database match
Adduct	Adduct type of ion of tentative database match
Matched Adduct	Monoisotopic mass of adduct of tentative database match
ppm	Mass accuracy of matched adduct against experimental *m/z*
Database	The unique HMDB identifier of tentative database match
Kingdom	Chemical taxonomy as given in HMDB database entry
Super Class	Chemical taxonomy as given in HMDB database entry
Class	Chemical taxonomy as given in HMDB database entry
Sub Class	Chemical taxonomy as given in HMDB database entry
Direct Parent	Chemical taxonomy as given in HMDB database entry

Features where no identification with ppm < 10 was achieved against the Human Metabolome Database are shaded in grey.

Supporting Data Files – FTIR and FIE-MS Metabolite Fingerprinting Data Sets

The following data files are provided for FTIR and FIE-MS metabolite fingerprinting data sets as processed spectra after total count normalisation in comma separated value (.csv) files in a combined ZIP archive file with Supporting Data Matrices 1 and 2:

File Name	Description
fiems_pl_neg_pa.csv	Negative Ion FIE-MS plasma data set labelled by participant
fiems_pl_neg_tr.csv	Negative Ion FIE-MS plasma data set labelled by activity
fiems_pl_pos_pa.csv	Positive Ion FIE-MS plasma data set labelled by participant
fiems_pl_pos_tr.csv	Positive Ion FIE-MS plasma data set labelled by activity
fiems_rs_neg_pa.csv	Negative Ion FIE-MS saliva data set labelled by participant
fiems_rs_neg_tr.csv	Negative Ion FIE-MS saliva data set labelled by activity
fiems_rs_pos_pa.csv	Positive Ion FIE-MS saliva data set labelled by participant
fiems_rs_pos_tr.csv	Positive Ion FIE-MS saliva data set labelled by activity
fiems_st_neg_pa.csv	Negative Ion FIE-MS stool data set labelled by participant
fiems_st_neg_tr.csv	Negative Ion FIE-MS stool data set labelled by activity
fiems_st_pos_pa.csv	Positive Ion FIE-MS stool data set labelled by participant
fiems_st_pos_tr.csv	Positive Ion FIE-MS stool data set labelled by activity
ftir_pl_pa.csv	FTIR plasma data set labelled by participant
ftir_pl_tr.csv	FTIR plasma data set labelled by activity
ftir_rs_pa.csv	FTIR saliva data set labelled by participant
ftir_rs_tr.csv	FTIR saliva data set labelled by activity
ftir_st_pa.csv	FTIR stool data set labelled by participant
ftir_st_tr.csv	FTIR stool data set labelled by activity
